# The impact of the COVID-19 pandemic on underrepresented early-career PhD and physician scientists

**DOI:** 10.1017/cts.2021.851

**Published:** 2021-09-14

**Authors:** Jamie M Doyle, Natalia E. Morone, Chelsea N. Proulx, Andrew D. Althouse, Doris M. Rubio, Maya S. Thakar, Audrey J. Murrell, Gretchen E. White

**Affiliations:** 1 Division of Clinical Innovation, National Center for Advancing Translational Sciences, Bethesda, MD, USA; 2 Boston Medical Center, Boston University School of Medicine, Boston, MA, USA; 3 Institute for Clinical Research Education, General and Internal Medicine, University of Pittsburgh School of Medicine, Pittsburgh, PA, USA; 4 Department of General Internal Medicine, University of Pittsburgh Graduate School of Public Health, Pittsburgh, PA, USA; 5 Department of Epidemiology, University of Pittsburgh Graduate School of Public Health, Pittsburgh, PA, USA; 6 School of Business, University of Pittsburgh, Pittsburgh, PA, USA

**Keywords:** COVID-19, diversity, career development, biomedical research workforce, physician-scientists, intervention

## Abstract

Underrepresented minorities have higher attrition from the professoriate and have experienced greater negative impacts of the COVID-19 pandemic. The purpose of this study was to compare the impact of COVID-19 on the lives of 196 early-career physician-scientists versus PhD researchers who are underrepresented in biomedical research. Participants in the Building Up study answered questions on the impact of the COVID-19 pandemic on their personal and professional lives, and a mixed-methods approach was used to conduct the analysis. While most participants experienced increases in overall stress (72% of PhD researchers vs 76% of physician-scientists), physician-scientists reported that increased clinical demands, research delays, and the potential to expose family members to SARS-CoV-2 caused psychological distress, specifically. PhD researchers, more than physician-scientists, reported increased productivity (27% vs 9%), schedule flexibilities (49% vs 25%), and more quality time with friends and family (40% vs 24%). Future studies should consider assessing the effectiveness of programs addressing COVID-19-related challenges experienced by PhD researchers and physician-scientists, particularly those from underrepresented backgrounds.

## Introduction

The COVID-19 pandemic exacerbated longstanding issues in public health and the biomedical research enterprise. As COVID-19 cases escalated during the Spring of 2020, research studies halted, laboratories temporarily shuttered, and many scientists pivoted their research to COVID-19 and addressing pressing public health needs brought by the pandemic [1]. Early-career scientists faced additional challenges. Research productivity, mentoring, and professional development are critical for early-career faculty trying to obtain tenure and promotion, yet these activities were also limited or halted during the early months of the pandemic [1–3]. Moreover, when compared to more senior faculty, early-career researchers are more likely to have caring responsibilities (40% vs 67%, respectively [1]) and higher teaching loads, including the time-consuming switch to online instruction [4].

The disruptions created by the COVID-19 pandemic are compounded for early-career faculty, particularly those from academic medical centers. Early-career physician-scientists faced unique challenges during the pandemic, with many being redeployed to clinical duties at the expense of their research [3]. At the same time that research studies were halted, physicians had increased clinical hours and/or saw patients via telehealth [5,6]. Physicians responded to the pandemic by working more shifts, working longer hours, adding COVID-19 specific outpatient clinics to their schedules, and covering for their colleagues when they fell ill with COVID-19.

The COVID-19 pandemic also intensified preexisting inequities in academia [7,8]. Underrepresented minorities have higher attrition from the professoriate and have experienced greater negative impacts from the pandemic as compared to others [2,9,10]. Early-career faculty of color are also disproportionately affected by the “Minority Tax” where they spend more time participating in diversity efforts than their well-represented peers, detracting from time that could be spent conducting research and writing grants, which are essential for promotion and obtaining tenure [11–14]. Underrepresented physician-scientists are also more likely to be assigned to patient care duties and community service, often in underserved or poor communities without large academic medical centers [14]. While it is clear that the COVID-19 pandemic has strained early-career faculty, particularly those who are underrepresented, it is not clear whether the pandemic has differentially impacted early-career physician-scientists and PhD researchers. Therefore, using a mixed-methods approach, the purpose of this study is to compare the impact of the COVID-19 pandemic on early-career physician-scientists and PhD researchers who are underrepresented in biomedical research.

## Methods

Data for this study came from the baseline survey of participants in the Building Up a Diverse Workforce for Biomedical Research Trial (Building Up). Building Up is a cluster randomized trial at 25 Clinical and Translational Science Award institutions. Sites were randomized to one of two interventions for postdoctoral fellows and junior faculty underrepresented in health-related sciences [15]. Both interventions lasted 10 months and included varying intensities of four components (i.e., mentoring, monthly sessions, networking, and coursework). A single institutional review board at the University of Pittsburgh approved the protocol, and participants gave electronic informed consent. Participants completed an online baseline survey with structured and open-ended questions, including questions that assessed the impact of COVID-19 on participants’ home life and research, in September 2020 or October 2020. Only baseline data were used for this analysis.

### Survey Measures and Qualitative Data

Participants answered questions on the impact of the COVID-19 pandemic on their personal and professional lives. These questions assessed whether changes to their home life due to the COVID-19 pandemic impacted their ability to work, whether the COVID-19 pandemic impacted their ability to conduct research, and what in their life had changed since the COVID-19 pandemic began. Participants were also asked to respond to two open-ended questions: “How has the COVID-19 pandemic impacted your career trajectory?” and “Is there anything else you want us to know about how the COVID-19 pandemic has impacted your professional/academic life (either positively or negatively) in the past 6 months?” A full list of survey questions is in Supplemental Table 1.

### Analysis

Differences in the impact of COVID-19 by career type (i.e., physician scientist vs PhD researcher) were tested with the Chi-square or Fisher’s exact test for categorical variables and the Cochran Armitage test for ordinal variables. We used Fisher’s exact test when the expected cell counts were <5. The following *P*-values were Fisher’s exact: increased free time and increased discrimination. All reported *P*-values were two-tailed, and *P*-values < 0.05 were considered statistically significant. Survey measures were analyzed using SAS version 9.4 (SAS Institute, Cary, NC, USA).

A thematic, qualitative analysis of responses to both open-text questions was also conducted. A draft codebook was developed inductively for each question by the primary coder (CNP). Primary and secondary (MST) coders reviewed the initial codebook to ensure comprehensive and distinct definitions. Once finalized, the primary and secondary coders applied the codebooks to each participant response. To assess interrater reliability, Cohen’s Kappa was calculated for the application of each codebook. The average Kappas showed substantial agreement [16], with 0.71 for the question, “How has the COVID-19 pandemic impacted your career trajectory?” and 0.72 for the question, “Is there anything else you want us to know about how the COVID-19 pandemic has impacted your professional/academic life (either positively or negatively) in the past 6 months?” Disagreements were adjudicated by both coders prior to thematic analysis. The thematic analysis for this paper was guided by different impacts experienced by physician-scientists and PhD researchers in line with our research question.

## Results

### Quantitative Results

**Fig. 1.**
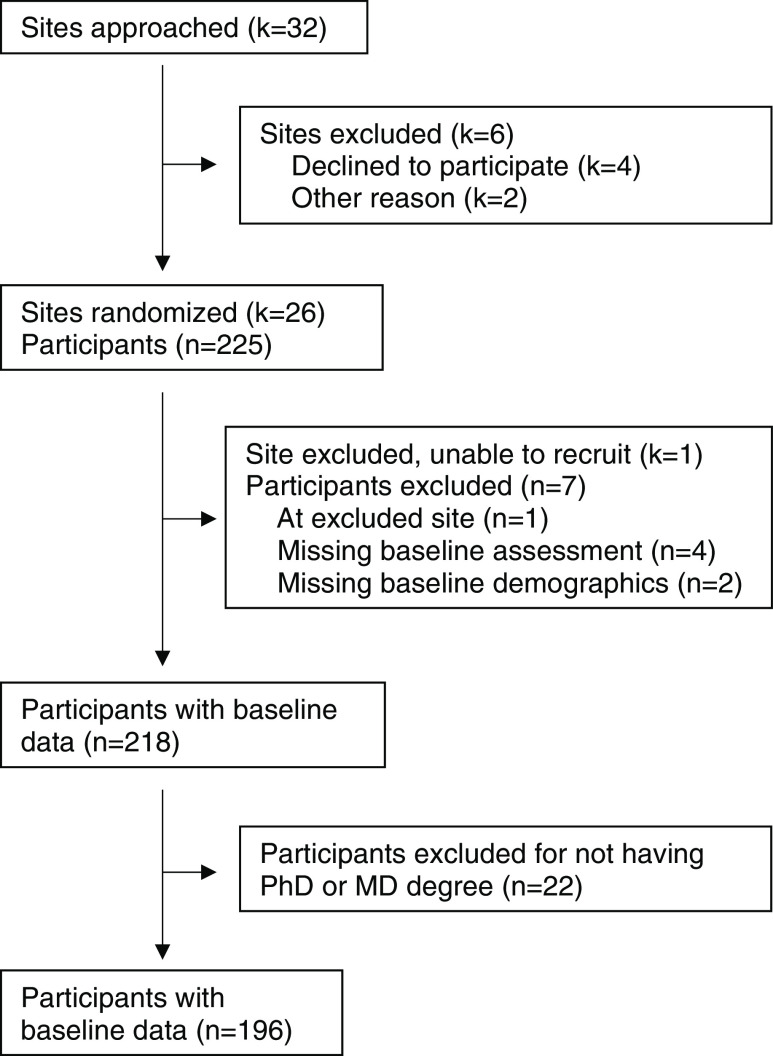
Institution and participant flow diagram.

Table 1 displays the demographic characteristics and the career status of survey respondents, and Fig. 1 shows a flow diagram of the final sample of 196 individuals. Too few participants had an MD/PhD to analyze separately and were excluded from analyses. The median age of PhD researchers and physician-scientists was 36 years, and most were women (78.3% and 86.6%, respectively). Approximately one-third of PhD researchers and physician-scientists were Hispanic, and one-third were Black or African American.

**Table 1.** Characteristics of early-career PhD researchers and physician scientists in the Building Up a Diverse Workforce for Biomedical Research Trial

	PhD researcher (n = 129)		Physician scientist (n = 67)	
Characteristic	n	(%)^a^	n	(%)^a^
Age (median, 25th–75th percentile)	36	32–40	36	33–38
Gender				
Male	27	20.9	9	13.4
Female	101	78.3	58	86.6
Gender minority	1	0.8	0	0.0
Race/Ethnicity				
Hispanic/Latinx	42	32.6	25	37.3
Non-Hispanic/Latinx				
White	18	14.0	8	11.9
Black	42	32.6	21	31.3
Asian	17	13.2	7	10.5
Other^b^	10	7.8	6	9.0
Career status				
Postdoctoral fellow	67	51.9	27	40.3
Faculty	62	48.1	40	59.7

a Unless otherwise specified.

b Middle Eastern/North African or multirace.

The impact of COVID-19 on PhD researchers and physician-scientists is described in Table 2. Physician-scientists and PhD researchers reported similar percentages of disruptions to work (56.7% and 52.7%), difficulties concentrating (61.2% and 65.1%), and increased overall stress (76.1% and 72.1%) since the pandemic began. Significantly higher proportions of PhD researchers than physician-scientists reported increased productivity (27.1% vs 9.0%), more flexibility in schedule (48.8% vs 25.4%), and more quality time with friends and family (40.3% vs 23.9%). Physician-scientists also reported fewer positive personal and professional changes since the COVID-19 pandemic began.

**Table 2.** Impact of COVID-19 on early-career PhD researchers and physician scientists

	PhD researcher (n = 129)		Physician scientist (n = 67)		*P*-value
	n^a^	(%)	n^a^	(%)	
Changes in home life due to the COVID-19 pandemic have greatly impacted ability to work					0.89
Strongly agree	32	25.0	13	19.4	
Agree	35	27.3	25	37.3	
Neutral	25	19.5	17	20.9	
Disagree	23	18.0	7	10.5	
Strongly disagree	13	10.2	8	11.9	
COVID-19 pandemic has impacted ability to conduct research					0.55
Strongly agree	35	27.1	25	37.3	
Agree	56	43.4	20	29.9	
Neutral	11	8.5	9	13.4	
Disagree	18	14.0	10	14.9	
Strongly disagree	9	7.0	3	4.5	
Changes since the COVID-19 pandemic began					
Continued to work even though in close contact with people who might be infected	18	14.0	46	68.7	<0.001
Disruptions to work	68	52.7	38	56.7	0.59
Lack of equipment or resources to work efficiently and effectively	32	24.8	13	19.4	0.39
Increased workload or work responsibilities	60	46.5	31	46.3	0.97
Decreased workload or work responsibilities	15	11.6	3	4.5	0.10
Increased productivity	35	27.1	6	9.0	0.003
Difficulties concentrating	84	65.1	41	61.2	0.60
Increased financial stress	41	31.8	14	20.9	0.11
Increased overall stress	93	72.1	51	76.1	0.54
Increased free time	8	6.2	4	6.0	0.95
More flexibility in schedule	63	48.8	17	25.4	0.002
Increased discrimination	9	7.0	5	7.5	0.99
Strengthened relationships with others	26	20.2	12	17.9	0.71
More quality time with friends and family	52	40.3	16	23.9	0.02
Other	18	14.0	8	11.9	0.69
Number of negative changes since the COVID-19 pandemic began^b^					0.12
0	5	3.9	3	4.5	
1	16	12.4	6	9.0	
2	20	15.5	6	9.0	
3	34	26.4	15	22.4	
4	26	20.2	19	28.4	
5	19	14.7	9	13.4	
6	5	3.9	4	6.0	
7	2	1.6	5	7.5	
8	2	1.6	0	0.0	
Number of positive changes since the COVID-19 pandemic began^c^					<0.001
0	31	24.0	31	46.3	
1	44	34.1	19	28.4	
2	26	20.2	13	19.4	
3	17	13.2	3	4.5	
4	5	3.9	1	1.5	
5	3	2.3	0	0.0	
6	3	2.3	0	0.0	

a Numbers may not add up to total due to missing values.

b Includes continued to work even though in close contact with people who might be infected, disruptions to work, lack of equipment or resources to work efficiently and effectively, increased workload or responsibilities, difficulties concentrating, increased financial stress, increased overall stress, and increased discrimination.

c Includes decreased workload or responsibilities, increased productivity, increased free time, more flexibility in schedule, strengthened relationships with others, and more quality time with friends and family.

### Qualitative Data Results

**Theme 1: The perceived reasoning behind research suspension and its effects differed between PhD researchers and physician-scientists.**

Most participants described some degree of research delay or suspension because of the pandemic. Both PhD researchers and physician-scientists often attributed these suspensions to research shutdowns in response to pandemic mitigation efforts. However, physician-scientists also reported that increased clinical demands related to the pandemic delayed their research projects. Research suspension had similar negative impacts for both groups, including challenges to networking and collaboration, and increases in stress and anxiety.

Positive effects of research suspension varied by career path. Despite shutdowns, physician-scientists reported that they were able to expand their research interests and collaborations into COVID-related areas, although not always by choice, as one physician scientist mentioned, “my primary research focus has been placed on hold while I have been to some extent forced into taking COVID research and increased clinical responsibilities surrounding it.” Relatively few participants explained that suspended research increased their productivity or free time, but all who did were PhD researchers. For these individuals, time usually dedicated to actively running a research study was now available to write manuscripts and prepare grant proposals. Childcare responsibilities while working remotely conflicted with productivity for most participants. However, PhD researchers were more likely to reflect on the positive aspects of increased quality time with family and children than physician researchers. For example, one participant noted:

*“I have had more family time, which has decreased stress. Research takes a great deal of work and family becomes resentful when I'm not home. Since the pandemic, I have been with them more and our family dynamic has greatly improved.” – PhD researcher*

**Theme 2: Institutional actions, such as hiring freezes and research shutdowns, had a more profound impact on employment and financial insecurity for PhD researchers than physician-scientists.**

PhD researchers frequently mentioned that pandemic-related institutional policies such as hiring freezes and changes to tenure and promotion created employment and financial insecurity. Concerns about the future availability of academic jobs were most prevalent among PhD researchers, as one participant noted:

*“The job market for assistant professors at research universities is very poor due to hiring freezes. I still have time on my postdoctoral fellowship, but I do not know if there will be many jobs to even apply to once everything is over.” – PhD researcher*

Physician-scientists mentioned financial stress and employment insecurity less often than PhD researchers, possibly due to increased clinical demands. For example, one physician scientist described being able to supplement an institutional salary cut through moonlighting but at the expense of their well-being:

*“To mitigate for financial losses, my university cut salaries across the board by 20%…I began moonlighting in the COVID ICU to make up for this deficit. The fatigue in doing so impacted my research productivity and well-being for some time. I am grateful that I have the skills and training to be able to close this gap financially for my family.” – Physician scientist*

Physician-scientists who did describe employment insecurity expressed worry over the availability of research funding, their ability to compete for funding, and availability of protected research time more so than the availability of tenure-track faculty positions.

**Theme 3: PhD researchers and physician-scientists reported different sources of pandemic-related psychological distress.**

Participants described increased psychological distress, including more stress, burnout, and anxiety, as well as less concentration and motivation. For PhD researchers, psychological distress was primarily attributed to suspended research activities, employment insecurity, and childcare disruptions. In addition to these factors, physician-scientists were distressed by increased clinical demands and the potential to expose family members to SARS-CoV-2. The physician scientist below described the impact of the added stress on their ability to conduct research:

*“As a clinician, my stress level is much higher and I am closer to burn out than I ever thought possible. Working is exhausting and trying to work from home also exhausting. I am able to complete short tasks but have been so limited in the amount of sustained focus that is needed to accomplish relevant research.” – Physician scientist*

Whether physician-scientists experienced increased clinical demands depended on their specialty. For example, one surgical resident found that their clinical loads decreased significantly giving them the opportunity to *“rediscover a lot of my motivation, creativity, and intellectual creativity.”*

## Discussion

PhD researchers and physician-scientists underrepresented in biomedical research experienced a wide breadth of impacts from the COVID-19 pandemic, and this study found similarities and differences between these groups. Most PhD researchers and physician-scientists experienced disruptions to work, difficulties concentrating, and increases in overall stress, due to the COVID-19 pandemic, but there were unique challenges differentiating the groups. Physician-scientists reported that increased clinical demands delayed their research and the potential to expose family members to SARS-CoV-2 also caused psychological distress. Clinicians separated themselves from their family members to avoid potentially exposing them to the virus, which further placed stress on the clinician and their family [17,18]. These findings are consistent with those from McCormack and colleagues showing that, within one month of the COVID-19 pandemic in the United States, increased clinical time hindered research efficiency and focus for early-career physician-scientists [19]. However, findings from the qualitative analysis suggest that this likely differs by subspecialty as some subspecialties, such as surgery, saw many elective procedures canceled during the early days of the pandemic [20].

While there were no statistical differences in financial stress between PhD researchers and physician-scientists reported in the quantitative analysis, the qualitative findings suggest that PhD researchers may have experienced greater personal financial insecurity. As one physician scientist communicated, physicians may have more employment stability and can moonlight to supplement financial deficits given the high demand for clinicians to care for COVID-19 patients. However, this came at the cost of possible burnout, whereas most PhD researchers were unable to supplement their incomes through direct patient care. PhD researchers were also particularly concerned about future employment due to hiring freezes instituted by universities during the pandemic [20–22]. This concern is widespread among early-career researchers as nearly two-thirds of postdoctoral researchers in one survey felt that the COVID-19 pandemic would negatively affect their career prospects [23]. Since PhD researchers and physician-scientists from underrepresented backgrounds left the biomedical research workforce at higher rates before the COVID-19 pandemic began [24], the collateral damage of the COVID-19 pandemic on goals to diversify the biomedical research workforce could reverberate for years after the pandemic ends as women and minorities, lacking the resources and support to remain in research careers, leave permanently [25].

Physician-scientists also experienced fewer positive impacts on their career and personal lives since the COVID-19 pandemic began as compared to PhD researchers. The pandemic likely exacerbated existing issues among physician-scientists. For example, historically, physician-scientists had unique challenges with identifying approaches to balance work/life demands, time-consuming requirements to maintain clinical credentials, and difficulty finding mentors [26]. Thus, challenges balancing work and life demands and identifying mentors were made more difficult during the pandemic [3]. While many PhD researchers were able to shift teaching and research responsibilities completely online, clinicians may not have had this flexibility. Coupled with the additional stress of potentially exposing family members to SARS-COV-2 [5,6], it is unsurprising that physician-scientists reported fewer positive impacts of the pandemic on their career.

Despite many differences in the impact of COVID-19 on their personal and professional lives, PhD researchers and physician-scientists also reported several similarities. Research suggests that there are five areas where early-career physician-scientists faced significant challenges during the COVID-19 pandemic: research productivity, funding, professional development, mentorship, and wellness [3]. Since baseline assessments for Building Up were administered prior to those publications, Building Up did not cover these five areas in detail with focused questions. However, both physician-scientists and PhD researchers frequently mentioned challenges related to productivity, funding transitions, and wellness in response to open-ended questions. This pattern is consistent with research by Termini and Traver [22], which suggests that all early-career scientists, such as postdoctoral fellows and junior faculty, face similar challenges. Interestingly, few participants in our study noted professional development or mentorship as salient challenges, whereas a previous study of early-career trainees found that 20% of respondents reported issues with mentor access as a result of the pandemic [19].

This study has several strengths, including a large cohort of PhD researchers and physician-scientists underrepresented in biomedical research. While it has been acknowledged that communities of color are disproportionately affected by the pandemic, this is one of the first studies to specifically examine the impact the pandemic on early-career scientists from underrepresented backgrounds. The mixed-methods approach is also a strength of this study as the qualitative analysis added nuance to the reported challenges beyond the quantitative analysis.

The strengths of this study should be viewed considering several limitations. First, results of this study cannot be generalizable to the entire biomedical research workforce. While the source population for the study draws from over 20 institutions from across the United States, it is not nationally representative and includes only participants in the Building Up a Diverse Workforce for Biomedical Research Trial. Moreover, we did not collect information on the type of research that participants conducted, which may have differed among PhD researchers and physician-scientists. Second, the data only represents the experiences of participants at a single point in time early during the pandemic. Improvements in COVID-19 prevention and treatment, and adaptations made by institutions in response to the pandemic may have alleviated some of the stress and work disruptions reported by participants. The baseline survey was administered 5−6 months after the pandemic began in the United States and many of the COVID-19 questions began with, “since the pandemic began” or focused on the pandemic more generically. Since the early days of the pandemic were different than the months that followed, it is possible that these findings do not generalize to different timepoints during the pandemic. Future studies should consider collecting these measures longitudinally to measure the lasting impact of the pandemic on this population. Third, the study did not specifically ask about the role of new technologies, which likely facilitated scientists work or virtual meetings which may have decreased commute time and freed up more time for research. However, no participants mentioned the role of new technologies in facilitating research when answering open-ended questions. Finally, this study does not include a comparison group of well-represented early-career researchers. Indeed, while patterns reported here are consistent with studies of COVID-19 on researchers, it is possible that those who are underrepresented in biomedical research are experiencing its effects more intensively, given that COVID-19 has disproportionately devastated minority communities.

In summary, this is the first study, to our knowledge, that compares the impact of the COVID-19 pandemic on early-career PhD researchers and physician-scientists who are historically underrepresented in biomedical research. While much of the research has focused on gender differences [27–29] and the impact on clinician workload [5,6], no study to date compares the experiences of researchers that have clinical commitments with those who primarily focus on biomedical research and are underrepresented in the professoriate. Future studies could consider assessing the effectiveness of programs proposed to address COVID-19-related challenges experienced by PhD researchers and physician-scientists, particularly those from underrepresented backgrounds[3,25].

## Supplementary material

For supplementary material accompanying this paper visit http://dx.doi.org/10.1017/cts.2021.851. click here to view supplementary material
