# Supplementary material for: The impact of the COVID-19 pandemic on underrepresented early-career PhD and physician scientists
Source: J Clin Transl Sci. 2021 Sep 14;5(1):e174. doi: 10.1017/cts.2021.851 (PMC8545843; doi:10.1017/cts.2021.851)
Supplement: Supplementary file 1 [file S2059866121008517sup001.docx]

**Supplemental Table 1. Impact of COVID-19 Survey Questions**

| **Question/Prompt** | **Response Option** |
| --- | --- |
| Changes to my home life due to the COVID-19 pandemic have greatly impacted my ability to work | Strongly Agree, Agree, Neutral, Disagree, Strongly Disagree |
| The COVID-19 pandemic has impacted my ability to conduct research | Strongly Agree, Agree, Neutral, Disagree, Strongly Disagree |
| Since the COVID-19 pandemic began, what has changed for you? | **Check all that apply**   - Had to continue to work even though in close contact with people who might be infected (e.g., customers, patients, co-workers) - Disruptions to work (e.g., childcare needs in home, helping child with schoolwork, caring for family members - Lack of equipment or resources (e.g., computers or Wi-Fi) to work efficiently and effectively - Increased workload or work responsibilities - Decreased workload or work responsibilities - Increased productivity - Difficulties concentrating - Increased financial stress - Increased overall stress - Increased free time - More flexibility in schedule - Increased discrimination - Strengthened relationships with others - More quality time with friends and family - Other |
| Number of positive changes since the COVID-19 pandemic began | **Calculated variable based on the number checked:**   - Decreased workload or responsibilities - Increased productivity - Increased free time - More flexibility in schedule - Strengthened relationships with others - More quality time with friends and family |
| Number of negative changes since the COVID-19 pandemic began | **Calculated variable based on the number checked:**   - Had to continue to work even though in close contact with people who might be infected (e.g., customers, patients, co-workers) - Disruptions to work (e.g., childcare needs in home, helping child with schoolwork, caring for family members - Lack of equipment or resources (e.g., computers or Wi-Fi) to work efficiently and effectively - Increased workload or work responsibilities Difficulties concentrating - Increased financial stress - Increased overall stress - Increased discrimination |

These questions were developed as a part of the National Research Mentoring Network COVID-19 working group with funding from the National Institute of General Medical Sciences (NIGMS).
